# A case of enteropathy-associated T-cell lymphoma (Type I) arising in stomach without refractory celiac disease

**DOI:** 10.1186/1746-1596-7-172

**Published:** 2012-12-07

**Authors:** Liang Wang, Yang Liu, Xu-Yong Lin, Juan-Han Yu, Yuan Miao, Xue-shan Qiu, En-Hua Wang

**Affiliations:** 1 Department of Pathology, the First Affiliated Hospital and College of Basic Medical Sciences, China Medical University, Shenyang, 110001, China; 2 Institute of Pathology and Pathophysiology, China Medical University, Shenyang, 110001, China

**Keywords:** Enteropathy-associated T-cell lymphoma, Ulcer, Diagnosis, Stomach

## Abstract

Enteropathy-associated T-cell lymphoma (EATL) is a rare peripheral T-cell lymphoma which was classified into 2 types based on histology. EATL is often, but not always, associated with celiac disease. EATL type I is a large cell lymphoma which is more common in frequency and highly associated with celiac disease compared with type II. Jejunum and ileum are the common sites, although EATL can rarely occur in the duodenum, stomach and colon or outside the gastrointestinal tract. We herein presented one case of gastric EATL, which happened in a 73-year-old Chinese male patient. Histologically, the tumor was composed of polymorphic (pleomorphic, anaplastic, immunoblastic) lymphoid cells and numerous inflammatory cells, including histiocytes, neutrophils and eosnophils in the background. The pleomorphic lymphoid cells were diffuse and strongly positive for CD3 and partially positive for CD30, while negative for CD4, CD5, CD8 or CD56. The gastric EATL should be distinguished from other gastric lesions, such as peptic ulcer, poorly-differentiated adenocarcinoma and other types of lymphoma.

**Virtual slides:**

The virtual slide(s) for this article can be found here: http://www.diagnosticpathology.diagnomx.eu/vs/1174320824810970

## Background

Enteropathy-associated T cell lymphoma (EATL) is a rare primary extranodal T cell lymphoma which was thought to arise from the intraepithelial cells of the small intestines [1]. Based on the criteria of World Health Organization (WHO), EATL was divided into two subtypes. EATL type I is usually associated with refractory celiac disease and covers 80-90% of all EATL cases. This type frequently has large-cell or pleomorphic cytology and seldom expresses CD8 and CD56. EATL type II is sporadic, seldom associated with celiac disease, and covers 10-20% of all EATL cases and is characterized by monomorphic cytology with frequent expression of CD8 and CD56 [2]. Although EATL seldom occurs in stomach, it should be considered in the differential diagnosis when the lesion exhibits neoplastic lymphoid cells with infiltration background. In some cases, these inflammatory cells may be so abundant as to obscure the relative small number of tumor cells creating great diagnostic confusion with peptic ulcer. Considering the morphology of EATL, it should also be distinguished with other gastric tumors and lesions, such as peptic ulcer, poorly-differentiated adenocarcinoma and other types of lymphoma (especially diffuse large B-cell lymphoma and anaplastic large cell lymphoma).

## Case presentation

### Clinical History

A 73-year-old man presented with epigastric and humeral back intermittent colic pain, nausea, and epigastric fullness of 2 months’ duration. Physical examination showed tenderness in upper central abdomen just below the xiphoid process. Hematological and chemical studies gave normal results. Conventional ultrasonography revealed an ulcer (30×50 mm) at the greater curvature to anterior wall of the stomach. The computed tomography scan showed no obvious lesions in the spleen, pancreas, and kidneys. The patient had no history of refractory celiac disease. The patient underwent biopsy twice under gastroscope, while the result was not satisfied due to too much necrosis. We could not rule out the possibility of malignancy according to the result of ultrasonography and computed tomography scan. The patient desired to undergo surgery on his own initiative, and subtotal gastrectomy was performed. The patient was alive with no tumor recurrence or metastasis within 3 months of follow-up.

### Gross features

Grossly, the resected specimen contained a centrally ulcerated mass (30×50×20 mm) at the greater curvature to anterior wall of the stomach. The ulcer was irregular in shape, upheaved in edge and covered by the yellow-tan exudate.

### Microscopic features

**Figure 1:**
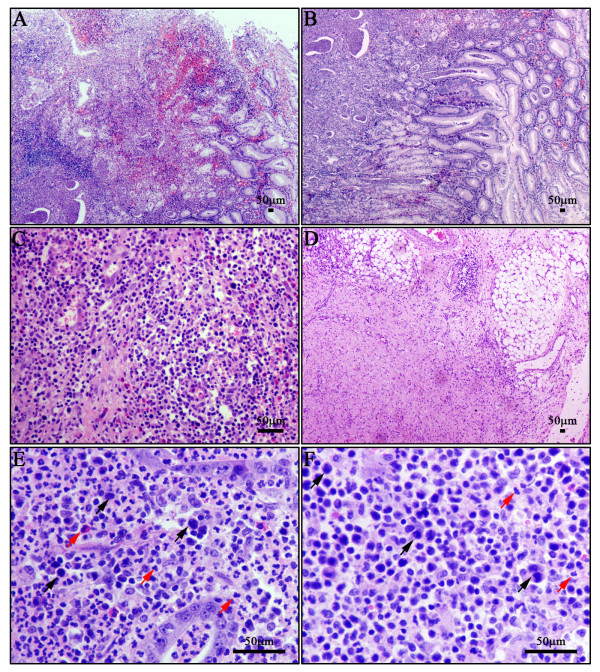
**Histological features of this case. A**: The tumor formed an ulcerating mucosal mass. **B**: Intraepithelial lymphocytosis was also seen in the adjacent gastric mucosa. **C** and **D**: The ulcer was deep and caused the transmural infiltration by florid mixed inflammatory cells. **E** and **F**: Neoplastic lymphoid cells (black arrow) were scattered amidst the inflammatory infiltrate. A large number of neutrophils and eosinophils were also observed (red arrow).

The tumor formed an ulcerating mucosal mass that invades the wall of stomach (Figure 1A-F). The ulcer was deep and caused the transmural infiltration by florid mixed inflammatory cells which were also seen in the lamina propria of adjacent non-ulcerated mucosa (Figure 1A-D). However, careful scrutiny revealed varying numbers of neoplastic lymphoid cells amidst the inflammatory background. The tumor cells exhibited various cytological appearances with concentrated chromatin. The tumor consisted of medium to large pleomorphic cells with round or angulated nuclei. Nucleoli were barely seen. A large number of histiocytes, neutrophils and eosinophils formed the inflammatory background. In addition, crypt abscesses and atypical hyperplasia of propria glands were also presented in this case (Figure 1E-F).

### Immunohistochemistry

**Figure 2:**
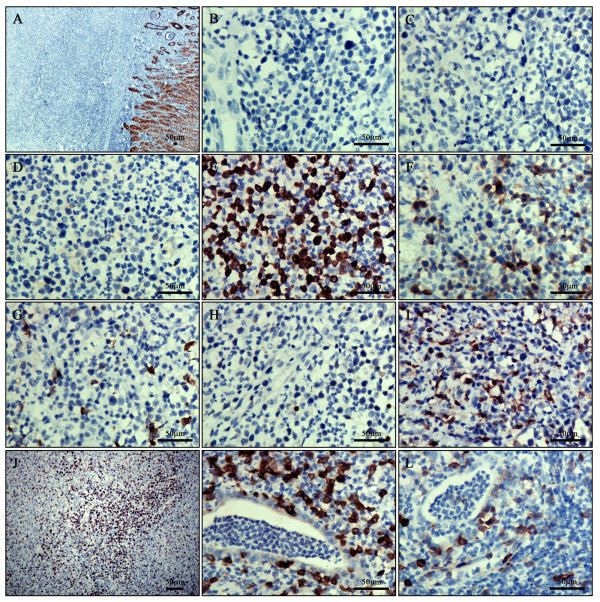
**Immunohistochemical staining. A**: The epithelium of gland was positive staining for AE1/AE3. **B**-**D**: The tumor cells were negative staining for CD5, CD8 and CD56. **E**: The tumor cells were strongly diffuse positive staining for CD3. **F**: The tumor cells partially expressed CD30. **G** and **H**: CD20 and Pax-5 labeled the scattered B cells amidst the inflammatory infiltrate. **I**: The histiocytes among the inflammatory infiltrate were positive for CD68. **M** and **N**: CD31 and CD34 underlined the rich vascular channels, whereas the histiocytoid cells were negative. **J**: Ki67 index was about 20%. **K** and **L**: The intraepithelial lymphocytes in the adjacent mucosa were positive for CD3 and CD30.

The immunohistochemical study showed that the pleomorphic lymphoid cells were negative for AE1/AE3, CD5, CD8, CD56 (Figure 2A-D) and CD4, strongly positive for CD3 (Figure 2E). The tumor cells were partially positive for CD30 (Figure 2F). CD20 and Pax-5 labeled the scattered B cells amidst the inflammatory background (Figure 2G and H). The histiocytes were labeled by CD68 (Figure 2I). Ki67 index was about 20% (Figure 2J). The intraepithelial lymphocytes in the adjacent mucosa shared the identical immunophenotype with the pleomorphic tumor cells (Figure 2K and L). In addition, the EBER in situ hybridization stain for EBV was negative. The results were listed in Table 1.

**Table 1:** Panel of immunohistochemical stains

**Immunohistochemical stain**	**Result**
Pan-cytokeratin (AE1/AE3)	-
CD3	+
CD4	-
CD5	-
CD8	-
CD56	-
CD30	+, varying proportion
CD20	-
Pax-5	-
CD68	-
Ki67	approximately 20%
the EBER in situ hybridization stain for EBV	-

## Discussion

EATL is a rare type of peripheral T-cell lymphomas in gastrointestinal tract and seldom occurs in stomach. O’Farrelly *et al.* firstly proposed the term EATL owing to the close association of this lymphoma with villous atrophy of the jejunal mucosa adjacent to EATL in 1986 [3]. EATL accounts for less than 1% of non-Hodgkin’s lymphoma and was most common in Europe, followed by North America and Asia [4]. The small intestine is the most common site of involvement. Presentation in the duodenum, stomach, colon or outside the gastrointestinal tract may occur but is rare [5,6]. Delabie *et al.* has reported approximately 8% of the EATL arising in stomach [7]. In spite of its low prevalence, EATL is one of the main causes of death in patients with long-lasting untreated refractory celiac disease, due to its aggressive nature and unresponsiveness to therapies currently available. Most of the lymphomas occur in stomach are of B cell origin [8-10]. Therefore, B cell lymphomas and other rare T cell lymphomas should be excluded when the diagnosis of gastric EATL is made. In addition, peptic ulcer and poorly-differentiated adenocarcinoma should also be considered in the differential diagnosis.

Although EATL has two subtypes with different histological features and mimics various gastric neoplasms, it can still be recognized or suspected on morphologic grounds. Histologically, the tumor forms an ulcerating mucosal lesion that invades the wall of gastrointestinal tract. Most commonly, the tumor cells are relatively monotonous, medium to large in size, with round or angulated vesicular nuclei, prominent nucleoli. The cytoplasm is moderate to abundant and pale-staining. Less commonly, the tumor exhibits marked pleomorphism with numerous inflammatory cells in the background. In the monomorphic form of EATL type II, the neoplastic cells have medium-sized round, darkly staining nuclei with a rim of pale cytoplasm. An inflammatory background is absent, and necrosis is usually less evident than in classical EATL type I. Immunohistochemically, the tumor cells are CD3+, CD4-, CD5-, CD7+, CD8−/+, CD103+, TCRβ+/−, and contain cytotoxic granule associated proteins. In almost all cases, a varying proportion of tumor cells express CD30. In contrast to EATL type II, EATL type I seldom expresses CD8 and CD56.

The differential diagnosis includes peptic ulcer, poorly differentiated adenocarcinoma, B cell lymphomas and other types of T cell lymphomas that present in the stomach. In some cases of EATL, the inflammatory cells may be so abundant as to obscure the relatively small number of tumor cells and therefore resembles the lesion of peptic ulcer. However, the intraepithelial lymphocytes of peptic ulcer are polyclonal and typically show no atypia. The pleomorphic cells in EATL indicate the property of poorly differentiated adenocarcinoma, but negative for AE1/AE3 staining will be helpful to distinguish between them.

The most common types of lymphomas involving the stomach are of B cell origin, and include MALT lymphoma, diffuse large B cell lymphoma (DLBCL), mantle cell lymphoma, and occasionally, follicular lymphoma. Mantle cell lymphoma and follicular lymphoma often produce multiple polyp-like extensions of the mucosa (lymphomatoid polyposis), a gross appearance that is not seen in EATL. DLBCL and Burkitt lymphoma often produce large masses that may grossly resemble EATL. Occassionally, the tumor cells of EATL are immunoblast-like and mimic the cells of DLBCL, but they do not express B cell markers such as CD20 and Pax-5 which are always expressed in DLBCL (like other B cell lymphomas). Therefore, it is readily distinguishable by their immunophenotypic characteristics.

Extranodal NK/T cell lymphoma is a lymphoma of natural killer (NK) or T cell lineage that most commonly presents with a facial mass. A small percentage of patients may present with gastrointestinal involvement. Because the morphology of the tumor cells is so variable, it is important to consider extranodal NK/T cell lymphoma in all cases of aggressive extranodal lymphoma associated with vascular invasion and necrosis. The key diagnostic features of NK/T cell lymphoma are the demonstration of NK/T cell markers (CD2, cytoplasmic CD3, CD56, and cytotoxic granule proteins) and Epstein-Barr virus (EBV), which is uniformly present. In contrast, EATL is not associated with EBV infection and infrequently expresses CD56.

Gamma-delta T cell lymphoma most commonly involves the liver and spleen (hepatosplenic gamma-delta T cell lymphoma), but less commonly involves other areas in the gastrointestinal tract. The diagnosis of gamma-delta T cell lymphoma is usually made based upon biopsy specimens demonstrating infiltrating atypical lymphocytes that express CD2, surface CD3, CD7, CD56, and CD16, and do not express CD4, CD5, CD8 or B-cell surface markers. The atypical lymphocytes express gamma/delta T cell receptors. Like gamma-delta T cell lymphoma, EATLs express pan-T antigens (surface CD3+) and do not express CD4, CD5, or CD8. In contrast to gamma-delta T cell lymphoma, EATLs typically express CD103. The T cell receptor beta gene is clonally rearranged. In addition, EATL (type I) seldom express CD56. These properties will be helpful to distinguish between them.

Patients with anaplastic large cell lymphoma (ALCL) typically present with painless lymphadenopathy. The tumor is composed of large blastic cells with horseshoe-shaped nuclei, prominent nucleoli, with or without a prominent golgi complex (seen as a paranuclear clearing, or hof) in a cohesive growth pattern. By immunohistochemistry, homogeneous and strong expression of CD30 in a membrane/golgi pattern can be observed. There is also expression of T-cell antigens or no lineage-specific antigens as in the case of the null cell type. However, a subpopulation of EATL may resemble ALCL histologically and partly express CD30. ALCL is not associated with celiac disease-type symptoms or the presence of the intraepithelial lymphocytic infiltrates in the adjacent “normal” mucosa.

In this case, peptic ulcer, poorly differentiated adenocarcinoma and B cell lymphoma were easily excluded because of the monoclonal tumor cells which expressed only T cell markers. Although tumor cells showed necrosis, positive immunostaining for CD3 and heavy inflammatory background, the negative result of EBV detection and absence of CD56+ NK/T cells excluded the possibility of extranodal NK/T cell lymphoma and Gamma-delta T cell lymphoma. ALCL was the most difficult one to be distinguished among the differential diagnosis. However, the intraepithelial lymphocytic infiltration and partly expression of CD30 were critical to rule out the diagnosis of ALCL. In addition, the pleomorphology and negative staining for CD56 and CD8 led to the diagnosis of EATL type I, but not type II. Taken these together, our final diagnosis was gastric EATL type I.

We herein reported a case of EATL arising in stomach which was an uncommon site for this disease. It worth noting that the severe necrosis and inflammatory background, which were also observed in other extranodal T-lymphomas [5,6,11], may obscure the relatively small number of tumor cells. For the same reason, the patient in this study underwent biopsy twice under gastroscope, while the result was not satisfied because of too much necrosis. In this situation, it was dangerous to make the diagnosis of gastritis or ulcer without careful evaluation of the lymphoid component. The final diagnosis in this case also underlined this point. In addition, as same as other T-lymphomas occurred in uncommon sites [12-16], the gastric EATL also exhibits the genetic disorder which will be helpful to distinguish from reactive process.

## Conclusion

This case indicates that EATL should be considered in the differential diagnosis of initial gastric lymphomas to prevent a wrong diagnosis of other intertia lymphomas. Thus, a better evaluation of the real frequency of occurrence needs to be established for this highly aggressive malignant tumor in the stomach.

### Consent

Written informed consent was obtained from the patient for publication of this case report and accompanying images. A copy of the written consent is available for review by the Editor-in Chief of this Journal.

## Competing interests

The authors declare that they have no competing interests.

## Authors’ contributions

LW analyzed the data and wrote the manuscript as a major contributor. YL, XL, JY and YM helped to perform the immunochemical staining. XQ and EW helped to revise the discussion section of this manuscript. All authors have read and approved the final manuscript.
